# mHealth and eHealth for Obesity and Types 2 and 1 Diabetes

**DOI:** 10.1155/2016/9627602

**Published:** 2016-11-01

**Authors:** Gianluca Castelnuovo, Giancarlo Mauri, Kayo Waki

**Affiliations:** ^1^Psychology Research Laboratory, Istituto Auxologico Italiano IRCCS, Ospedale San Giuseppe, Verbania, Italy; ^2^Department of Psychology, Catholic University of Milan, Milan, Italy; ^3^Department of Informatics, University of Milano-Bicocca, Milan, Italy; ^4^University of Tokyo, Tokyo, Japan

Obesity and diabetes are universally recognized as multifactorial pathologies with a complex interaction between genetic, individual, and environmental factors. Clinical interventions, which typically focus on weight loss, reduction of obesity-related comorbidities, and change in dysfunctional behaviors, should be implemented in a multidisciplinary context with a clinical team composed of endocrinologists, nutritionists, dieticians, physiotherapists, psychiatrists, psychologists, and sometimes surgeons.

Significant limitations in the multidisciplinary chronic care management of obesity and diabetes concern costs and long-term adherence and compliance. mHealth (also m-health, mhealth, or mobile health) could be defined as the practice of medicine, public health, and clinical health psychology supported by mobile communication devices, such as mobile phones, tablet computers, PDAs, activity trackers, and other tools for health services and information. mHealth could be useful also for type 1 diabetes that often needs rigorous daily routines and an enduring self-management.

This issue features excellent articles, such as an interesting analysis of the opportunities of the activity monitors provided by M. Miyauchi et al. A. Booth et al. proposed a computer-based tool focused on self-management for people diagnosed with type 2 diabetes. Moreover, the important topic of patient engagement using new technologies is discussed by G. Graffigna et al. Opportunities of telemonitoring in the follow-up step of the weight reduction programs are provided by G. Stumm et al. Particular attention to usability issues in diabetes mHealth apps for the elderly is discussed by M. Isakovic et al. Finally the topics of personal health record and self-management support-coaching are proposed by M. van Vugt et al.

Care programs including the use of mHealth platforms and new technologies could overcome limitations connected to the traditional inpatient chronic care management by providing promising opportunities for enhancing weight reduction and reducing complications in terms of long-term efficacy and effectiveness across clinical, organizational, and economic perspectives.

New technologies can help clinicians and motivate patients in maintaining significant lifestyle behavior changes; improving health outcomes, quality of life, and well-being; and ensuring functional patient empowerment and engagement.

More research is needed, particularly in the cost-effectiveness field, where mHealth has to demonstrate its competitiveness in comparison with the traditional approaches.

*Gianluca Castelnuovo* *Gianluca Castelnuovo* *Giancarlo Mauri* *Giancarlo Mauri* *Kayo Waki* *Kayo Waki*

